# Neurotensin (8-13) and Neuromedin N Neuropeptides Radiolabelling with Copper-64 Produced on Solid or Liquid Targets

**DOI:** 10.3390/molecules29061390

**Published:** 2024-03-20

**Authors:** Diana Cocioabă, Alexandra I. Fonseca, Radu Leonte, Ivanna Hrynchak, Roxana Tudoroiu-Cornoiu, Sergio J. C. do Carmo, Bogdan Burghelea, Simona Băruță, Ana Rita Almeida, Radu Șerban, Anca Dinischiotu, Antero J. Abrunhosa, Dana Niculae

**Affiliations:** 1 Radiopharmaceutical Research Centre, Horia Hulubei National Institute for Physics and Nuclear Engineering (IFIN-HH), 077125 Măgurele, Romania; diana.cocioaba@nipne.ro (D.C.); roxana.cornoiu@nipne.ro (R.T.-C.); george.burghelea@nipne.ro (B.B.); simona.baruta@nipne.ro (S.B.); radu.serban@nipne.ro (R.Ș.); 2 Faculty of Physics, Doctoral School of Physics, University of Bucharest, 077125 Bucharest, Romania; 3 ICNAS Pharma, University of Coimbra, 3000-548 Coimbra, Portugal; alexandrafonseca@icnas.uc.pt (A.I.F.); ivanna.ua@icnas.uc.pt (I.H.); sergiocarmo@uc.pt (S.J.C.d.C.); ana.rita.almeida@icnas.uc.pt (A.R.A.); antero@pet.uc.pt (A.J.A.); 4 Faculty of Chemical Engineering and Biotechnologies, Doctoral School of Applied Chemistry and Materials Science, National University of Science and Technology Politehnica Bucharest, 011061 Bucharest, Romania; 5 Coimbra Institute for Biomedical Imaging and Translational Research, Institute for Nuclear Sciences Applied to Health (CIBIT/ICNAS), University of Coimbra, 3000-548 Coimbra, Portugal; 6 Faculty of Biology, Doctoral School of Biology, University of Bucharest, 050095 Bucharest, Romania; anca.dinischiotu@bio.unibuc.ro

**Keywords:** copper-64, solid target, liquid target, neuropeptides, theranostic

## Abstract

On the verge of a theranostic approach to personalised medicine, copper-64 is one of the emerging radioisotopes in nuclear medicine due to its exploitable nuclear and biochemical characteristics. The increased demand for copper-64 for preclinical and clinical studies has prompted the development of production routes. This research aims to compare the (p,n) reaction on nickel-64 solid versus liquid targets and evaluate the effectiveness of [^64^Cu]CuCl_2_ solutions prepared by the two routes. As new treatments for neurotensin receptor-overexpressing tumours have developed, copper-64 was used to radiolabel Neurotensin (8-13) and Neuromedin N. High-quality [^64^Cu]CuCl_2_ solutions were prepared using ACSI TR-19 and IBA Cyclone Kiube cyclotrons. The radiochemical purity after post-irradiation processing reached 99% (LT) and 99.99% (ST), respectively. The irradiation of a solid target with 11.8 MeV protons and 150 μAh led to 704 ± 84 MBq/μA (17.6 ± 2.1 GBq/batch at EOB). At the end of the purification process (1 h, 90.90% activity yield), the solution for peptide radiolabelling had a radioactive concentration of 1340.4 ± 70.1 MBq/mL (n.d.c.). The irradiation of a liquid target with 16.9 MeV protons and 230 μAh resulted in 3.7 ± 0.2 GBq/batch at EOB, which corresponds to an experimental production yield of 6.89 GBq.cm3/(g.µA)*_sat_*. Benefiting from a shorter purification process (40 min), the activity yielded 90.87%, while the radioactive concentration of the radiolabelling solution was lower (492 MBq/mL, n.d.c.). The [^64^Cu]CuCl_2_ solutions were successfully used for the radiolabelling of DOTA-NT(8-13) and DOTA-NN neuropeptides, resulting in a high RCP (>99%) and high molar activity (27.2 and 26.4 GBq/μmol for LT route compared to 45 and 52 GBq/μmol for ST route, respectively). The strong interaction between the [^64^Cu]Cu-DOTA-NT(8-13) and the colon cancerous cell lines HT29 and HCT116 proved that the specificity for NTR had not been altered, as shown by the uptake and retention data.

## 1. Introduction

Copper-64 is a radionuclide with great theranostic potential in nuclear medicine, considering its unique qualities. Its clinical utility in positron emission tomography imaging (PET) was demonstrated while ongoing studies investigated the most effective therapeutic applications based on this radionuclide. Depending on the clinical need, it serves in ionic form, as copper chloride, or linked to a biospecific peptide carrier. This radioisotope presents a complex decay scheme, which includes electron capture, beta minus, and positron emissions. The low-energy positrons emitted (17.49%, max 0.653 MeV, mean 0.278 MeV) are excellent for positron emission tomography imaging, especially due to a spatial resolution similar to fluorine-18, as determined by comparable average free travel distance of positrons (0.70 mm in the case of copper-64 and 0.69 mm in the case of fluorine-18) [1,2]. The lack of abundant gamma emissions (only 0.47%, 1.346 MeV) is also an advantage since they can cause interferences that impact the image quality, as in the case of other non-pure positron emitters such as bromine-76, yttrium-86, rubidium-82, and iodine-124 [3]. The β^−^ emission (38.48%, max 0.579 MeV, mean 0.190 MeV) offers the possibility for targeted radionuclide therapy with a highly localised dose, in situ visualised by the means of β^+^ (PET). Additionally, the electron capture events determine the emission of Auger electrons (Auger L 0.84 keV, 58.0% and Auger K 6.54 keV, 22.62%) which increases the radiotoxicity of the radioisotope when delivered inside the targeted cells, in particular when the emitting radioisotope is trapped in the cell nucleus [1,4].

Copper, as a bioelement, is involved in numerous metabolic processes; therefore, its trafficking, accumulation, and clearance are tightly controlled in normal health but are disturbed in disease states such as dementia, cancer, inflammation, nutritional abnormalities, and inherited diseases of copper metabolism [5,6]. Many cancer types exhibit increased intra-tumoral copper uptake and/or altered systemic copper distribution. The understanding that copper bioavailability serves as a limiting factor for multiple aspects of tumour progression, including growth, angiogenesis, and metastasis, has prompted the development of copper-specific chelators as therapies to inhibit these processes. The differentiated uptake of copper ions by normal and tumour cells creates the potential for copper-64 to image and quantify those processes and eventually exploit them for therapy [7].

Copper-64 can be administered as a [^64^Cu]CuCl_2_ solution, but it can also be carried by biologically active molecules (peptides, antibodies, etc.), to highlight specific oncological conditions or ensure the delivery of the radiotoxic compound for therapeutic purposes [8].

Commonly produced in a low to medium energy cyclotron by proton bombardment of a nickel-64 target through (p,n) nuclear reaction, copper-64 can be obtained using either a solid, metallic electrodeposited nickel-64 target or a liquid target, containing a dissolved nickel-64 salt (usually nitrate) solution [9,10].

Each of the two routes has both advantages and limitations; the choice depends on the particular needs of both the production and clinical sites involved, and also on the technical capabilities. While the solid target route leads to higher activity and high specific activity of [^64^Cu]CuCl_2_ solution, it requires a longer preparation time of the target and involves the use of high molar hydrochloric acid in the post-processing steps. With the liquid targets, there is no need for pre- or postprocessing of the target, and this route allows for an easier process automation; however, lower activity and also lower production yields are expected. The question is, if both routes are feasible for high-quality radiopharmaceutical preparation, especially when the resulting solutions are used for radiolabelling of specific targeting molecules. In our previous study we demonstrated the suitability of both liquid and solid target approaches to radiolabel antibodies and nanobodies with comparable specific activities and stability [11]. The present study was designed to optimise and compare the radiolabelling processes of neuropeptides using [^64^Cu]CuCl_2_ prepared by solid and liquid targets routes, respectively, and investigate the stability and affinity of the resulting radiolabelled peptides.

Neurotensin (NT) and neuromedin N (NN) are two related, biologically active peptides, synthesized by a common precursor in the mammalian brain and intestine/gut [12,13]. Their regulatory activity acts through specific G-protein-coupled neurotensin receptors [14]. Neurotensin stimulates the proliferation in vitro of tumour cell lines originating from the pancreas, prostate, astrocytes, and lung [15,16,17]. Neurotensin receptors were identified and localised in tumour cells of Ewing’s sarcomas, meningiomas, astrocytomas, medulloblastomas, medullary thyroid cancers, and small cell lung cancers [18]. A pseudopeptide, the smallest active fragment of neurotensin, Neurotensin(8-13) was developed to thwart the rapid in vivo degradation of full-chain peptide, bearing changes which stabilise the molecule against enzymatic degradation. NT(8-13) binds to the same sites, with a higher affinity than NT does [19]. NN is a good marker of human colon cancer, in addition to NT, and recent studies suggest it might exert an autocrine-positive effect on human colon cancer growth [20]. Using NT(8-13) and NN as carriers of copper-64, we aim to develop targeted radiopharmaceutical agents to diagnose and treat cancers overexpressing neurotensin receptors.

## 2. Results

### 2.1. Copper-64 Production and Post-Irradiation Processing via Solid Target (ST) System

**Figure 1:**
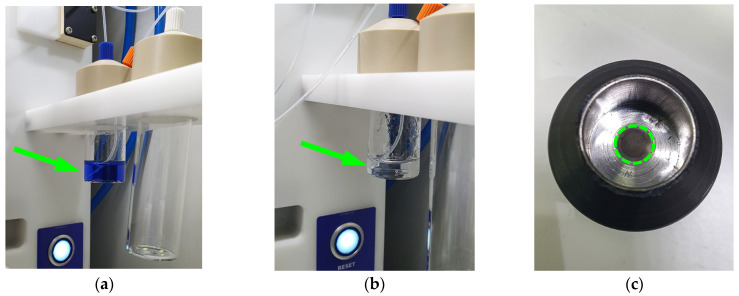
The electrodeposition process of nickel-64: (**a**) starting solution (blue); (**b**) final solution (colourless); (**c**) the resulted target (green circled) electrodeposited onto the irradiation shuttle.

Figure 1a,b shows the nickel-64 complex solution used for electrodeposition; the colour changed from intense blue to colourless, indicating the completion of the electrodeposition of the nickel target. The resulting target is a compact metallic round chip (Figure 1c), weighing 48 ± 1.3 mg, electrodeposited on the platinum support plate, part of the dedicated shuttle (which serves for both transport and irradiation purposes). The process step yield was 95–98%, determined by target weight compared with the mass of dissolved nickel.

Copper-64 (ST) production on the ACSI TR-19 cyclotron was achieved by increasing the irradiation parameters as compared to our previously reported work [21]: 150 μAh integrated beam current and higher extracted energy, of 14.2 MeV, corresponding to 11.8 MeV on target. Increasing the irradiation time from 4 h to 6 h, an average activity of 17.6 ± 2.1 GBq/batch (decay corrected at EOB) was obtained, slightly increasing the production yield from 435 ± 35 MBq/μA to 704 ± 84 MBq/μA. The activity produced by the integrated beam unit (μAh) was slightly increased, from 109 MBq to 117 MBq, respectively. Compared to Monte Carlo simulations, the experimental data were conforming with an acceptable deviation, determined by the particular geometry of the ST installation, and beam (de)focussing. The results are shown in Table 1.

The dissolution and purification process steps, carried out on EDS and TADDEO-PRF automated modules of the ALCEO system (Comecer), need 1 h to complete. The irradiated target was dissolved in 6 M HCl, and the resulting solution was loaded onto an AG1-X8 anion-exchange column. The target material, nickel-64, as well as the metallic impurities were washed from the column with 6 M and 4 M HCl. The final [^64^Cu]CuCl_2_ solution was obtained by eluting copper-64 from the cartridge with 0.5 M HCl. The total volume of the purified solution can be fractionated, by selecting the fraction with the highest radioactive concentration (5–10 mL), or it can be used in a larger volume (up to 20 mL). At the end of the process (1 h after EOB), the activity of the solution used for peptides radiolabelling was 16.2 ± 0.8 GBq, which is equivalent to an activity yield (AY) of 90.90 % (including decay and processing activity loss). These values correspond to 354.20 ± 1.95 MBq/mg of nickel-64 target.

### 2.2. Copper-64 Production and Post-Irradiation Processing via Liquid Target (LT) System

Copper-64 (LT) production on the IBA Cyclone Kiube Variable Energy cyclotron and the post-irradiation processing were previously published [11]. In brief, a 3–5 h irradiation time of ~100 mg enriched nickel-64 with 16.9 MeV protons on target (extracted energy 18 MeV) led to an average activity of 3.7 ± 0.2 GBq at EOB, (above 12 MBq/μAh).

The purification method, conducted on an IBA Synthera Extention module, resulted in less than 40 min to purified [^64^Cu]CuCl_2_ solution, with copper recovery yield of 94.15 ± 2.31%, corresponding to an activity yield (AY) of 90.87%.

### 2.3. Comparative Evaluation of the Neuropeptides Radiolabelling Using [^64^Cu]CuCl_2_ Prepared via ST and LT

The [^64^Cu]CuCl_2_ solutions produced by the irradiation of solid (ST) or liquid target (LT), respectively, were used to radiolabel the two neuropeptides derivatised with DOTA bifunctional chelator [2,2′,2″,2‴-(1,4,7,10—tetraazacyclododecane—1,4,7,10—tetrayl) tetra-acetic acid], namely DOTA-NT(8-13) and DOTA-NN. The solutions prepared by each of the methods have the following specifications: radiochemical purity 100%, determined by HPLC, radionuclidic purity ≥ 99.99%, determined by gamma spectrometry and half-life estimation by dose calibrator decay measurements, of 12.4 ± 0.2 h.

**Figure 2:**
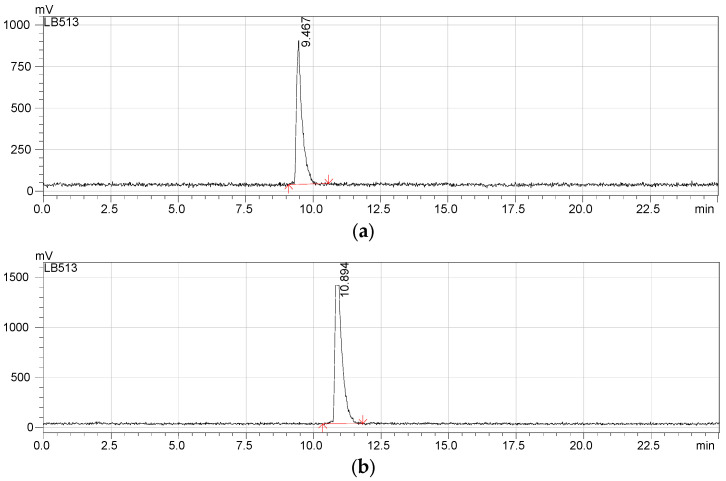
Radio-HPLC chromatograms of ST preparations of purified (**a**) [^64^Cu]Cu-DOTA-NT(8-13) solution and (**b**) [^64^Cu]Cu-DOTA-NN.

Both ST and LT production processes provided solutions that underwent similar radiolabelling procedures. The [^64^Cu]CuCl_2_ solutions were concentrated by evaporation and underwent pH adjustment to 3.8–4.0 with ammonium acetate before being used for peptide radiolabelling. Depending on the production method, different volumes of the concentrated [^64^Cu]CuCl_2_ purified solution were used for radiolabelling the same quantity of each neuropeptide (20 nmoles). Compared to the LT, which produced a solution with a lower radioactive concentration of 492.5 ± 9.8 MBq/mL, the labelling solution obtained using the ST had a concentration of 1340.4 ± 70.1 MBq/mL. Consequently, the volumes used for radiolabelling were 1.00 mL and 1.75 mL for ST and LT routes, respectively. The radiolabelling parameters for ST and LT experiments are presented in Table 2. The radiochemical purity (RCP) of the purified radiolabelled peptides [^64^Cu]Cu-DOTA-NT(8-13) and [^64^Cu]Cu-DOTA-NN was assessed by using two distinct radio-HPLC systems, Shimadzu Prominence 20 A for ST and Agilent 1260 Infinity II systems for LT; the columns and assay methods are described in detail in the Section 4. The radiochemical purity for both radiolabelled peptides was ≥ 99.99%, as indicated by the radio-HPLC chromatograms for the ST experiment (Figure 2a,b), where the corresponding retention times were t_R_ = 9.467 min for [^64^Cu]Cu-DOTA-NT(8-13) and t_R_ = 10.894 min for [^64^Cu]Cu-DOTA-NN, respectively.

**Figure 3:**
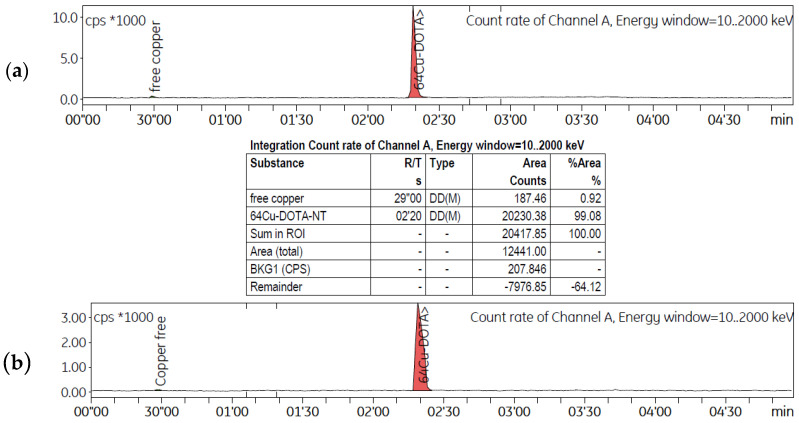

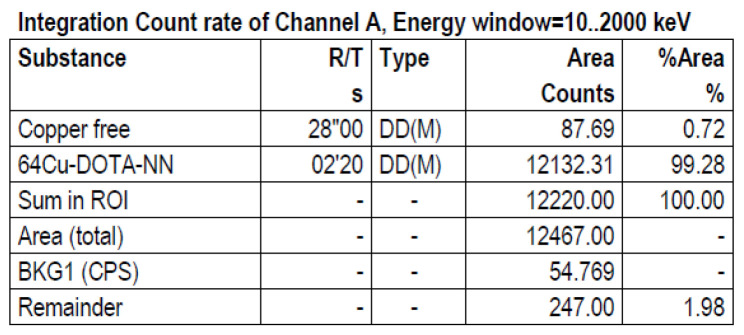
Radio-HPLC chromatograms of LT preparations of purified (**a**) [^64^Cu]Cu-DOTA-NT(8-13) solution and (**b**) [^64^Cu]Cu-DOTA-NN solution.

According to the analysis method used for LT experiments, the presence of small quantities of free copper-64 can be seen after the purification of the radiolabelled solution, signalled at the retention time t_R_ = 0.28 min, while the peak related to the radiolabelled peptides is highlighted at a retention time t_R_ = 2.20 min, as can be seen in Figure 3a,b. The chromatograms indicate that both radiolabelled peptides have a radiochemical purity higher than 99%.

Due to the higher radioactive concentrations employed in ST experiments, the radiolabelling resulted in slightly higher radiolabelling yields compared to LT experiments. However, the resulting radiopharmaceuticals have excellent RCPs, of over 99% and high molar activity of [^64^Cu]Cu-DOTA-NT(8-13) mean values being 27.2 and 45 GBq/μmol for LT and ST respectively, and of [^64^Cu]Cu-DOTA-NN, ranging from 26.4 to 52 GBq/μmol for LT and ST routes preparation, respectively.

### 2.4. In Vitro Study for Peptide Radiolabelling

**Figure 4:**
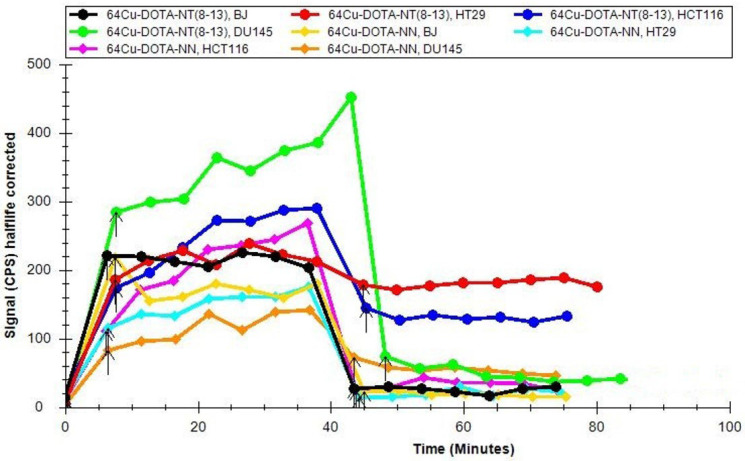
Overlay of the uptake–retention curves of the radiolabelled peptides on the cell lines.

We have established a real-time analysis of the peptide–receptor interaction, based on 7 data points (about 35 min) for an uptake assessment, and a minimum of 7 data points for a retention assay, based on our prior experiments [22]. The uptake profiles of the radiolabelled peptides to cancer cells show rapid binding in the first minutes after incubation with [^64^Cu]Cu-DOTA-NT(8-13) and [^64^Cu]Cu-DOTA-NN, respectively, but at different signal amplitudes, as presented in Figure 4. The high uptake of neurotensin derivative in the prostate (DU145) and colon (HT29 and HCT116) cancers was observed, and as such was the uptake of neuromedin peptide in the colon (HCT116), demonstrating that the radiolabelled peptides are suitable for the intended use. Furthermore, the high retention of [^64^Cu]Cu-DOTA-NT(8-13) in colon cancer cells (HT29 and HCT116) is promising for further investigations of this radiopharmaceutical for targeted theranostic. The uptake of the ^64^Cu-radiolabelled peptides in fibroblasts suggests passive uptake, confirmed by the lack of retention. A low or insignificant retention was also observed in the case of [^64^Cu]Cu-DOTA-NT(8-13) with DU145 and of [^64^Cu]Cu-DOTA-NN with HT29, HCT116, and DU145.

## 3. Discussion

Both solid target and liquid target routes, comparatively tested for the production of [^64^Cu]CuCl_2_ solution, present particular advantages that are important to consider for selecting one of them. While the ST route led to higher activities, specific activities, and higher efficacy of the irradiation process, in terms of activity produced per μA or μAh, the LT runs with similar AY. As an advantage, LT proved to be more time efficient and cleaner (due to an entirely closed process), and thus, could result in less exposure of the personnel. The irradiation processes were conducted on very performant cyclotrons, allowing for the selection of the optimal parameters and the best energy window of the nuclear reaction. Both routes are fully automatised and require post-irradiation purification and recovery of the highly enriched target material.

Compared with previously reported studies, the [^64^Cu]CuCl_2_ solutions were prepared with higher activities and in higher radioactive concentrations [11,21]. For ST, the radioactive concentration previously obtained was 90 ± 3.4 MBq/μAh (at EOB), while in this study we increased it to 117.3 ± 14 MBq/μAh (at EOB). For LT, the activity produced was increased from 2.8 ± 1.3 GBq (at EOB) to 3.7 ± 0.02 GBq, as reported in this study.

The quality of the solutions for the radiolabelling of peptides complies with the required specifications in terms of radiochemical and radionuclidic purities; after the necessary pH adjustment and concentration steps, they have comparable radioactive concentrations (1340 MBq/mL on ST vs. 492 MBq/mL on LT), which made possible a comparison of the radiolabelling capabilities. It is worth mentioning that except for the time needed for solid target preparation (15–20 h, which can be scheduled at any time before irradiation), the LT process duration is only 20 min shorter. The ST and LT share the disadvantage of expensive target material, while the total effectiveness of each process depends on particular setups and clinical needs.

At the end of the process (1 h in the case of ST and 40 min in the case of LT), the [^64^Cu]CuCl_2_ solutions were successfully used for the radiolabelling of DOTA-NT(8-13) and DOTA-NN neuropeptides, resulting in high RCP (>99%), and high molar activity (27.2 and 26.4 GBq/μmol for LT route compared to 45 and 52 GBq/μmol for ST route, respectively). Their specificity was tested using the Ligand Tracer method on different tumour cell lines and on fibroblasts. The strong interaction between the [^64^Cu]Cu-DOTA-NT(8-13) and the colon cancerous cell lines is proven by the uptake and retention data. After uptake stabilisation and washing with fresh medium, the peptide bonded to the receptors of the HT29 and HCT116 cell lines maintained the activity on cells at levels comparable with the uptake activities. The uptake of the peptides was also good for other peptide-cells combinations, when the washing caused the removal of a large portion of the radioactive solution from cellular membranes. The uptake profiles of radiolabelled peptides to cancer cells showed rapid binding in the first minutes after incubation with [^64^Cu]Cu-DOTA-NT(8-13) and [^64^Cu]Cu-DOTA-NN, respectively, and confirmed a nonspecific uptake in BJ fibroblasts.

## 4. Materials and Methods

### 4.1. Reagents and Equipment

The solid target material, isotopically enriched nickel-64 (99.53%) metallic powder was purchased from Isoflex Company (San Francisco, CA, USA). The reagents used for the preparation of the solid target were purchased as follows: ammonium chloride (≥99.9995% purity), nitric acid (67–69%), and water from Honeywell-Fluka, TraceSELECT (Muskegon, MI, USA); ultrapure hydrochloric acid and ammonium hydroxide (≥99.99%) from Sigma-Aldrich (Steinheim, Germany). The electrodeposition was performed on a dedicated shuttle manufactured by COMECER SpA (Castel Bolognese, Ravenna, Italy). In-house prepared columns loaded with AG1-X8 ion exchange resin from Bio-Rad Laboratories (Hercules, CA, USA) were used for purification.

For the liquid targets, enriched isotope nickel-64 was purchased from Fluidomica (Cantanhede, Portugal) in the form of metallic powder with >95.0% enrichment. Water (TraceSELECTTM, 99.9999999% metals basis) was obtained from Honeywell (Seelze, Germany). Ready to use CU Resin and anion exchange resin (SAX) were purchased from TrisKem International (Bruz, France).

The following were used in the peptide-labelling process: Neurotensin, short-chain 8-13, DOTA-NT(8-13) (purity 95%) from Eurogentec, Belgium; Neuromedin N, DOTA-NN (purity 95%) from PolyPeptide Group (Limhamn, Sweden); sodium chloride (0.9%) from Hemofarm, Serbia, ethanol (≥99.9%) from LiChrosol, Merck, Darmstadt, Germany; ultrapure water (resistivity 18.2 MΩcm/25 °C, TOC ≤ 5 ppb, pyrogenic impurities < 0.001 EU/mL) was freshly prepared with a Millipore Mili-Q Direct 8, from Millipore SaS, (Molsheim, France). The purification was carried out using a Strata-X 33 µm RP cartridge (60 mg/3 mL) from Phenomenex (Torrance, CA, USA), for the solid target experiment and a SPE Cartridge, Oasis HLB Plus Extraction from Waters Corporation, (Etten-Leur, The Netherlands) for the liquid target experiment, respectively.

The radiochemical purity (RCP) of the two radiolabelled peptides, [^64^Cu]Cu-DOTA-NT(8-13) and [^64^Cu]Cu-DOTA-NN, was evaluated with a liquid chromatography technique: radio-HPLC, using two different systems, equipped with a UV and gamma detector.

For the solid target experiment, HPLC analyses were carried out using a Shimadzu Prominence 20 A (Shimadzu, Kyoto, Japan) equipped with a Flowstar LB 513 detector of gamma radiation (Berthold Technologies GmbH & Co.KG., Bad Wildbad, Germany) and a C_18_ reverse-phase chromatography column (Waters SunFire, 4.6 × 150 mm, 3.5 μm). The analysis was performed using a gradient method at 1.0 mL/min flowrate. The solutions used as mobile phase were water with 0.1% (*v*/*v*) TFA, and acetonitrile with 0.1% (*v*/*v*) TFA.

For the liquid target experiment, a radio-HPLC, Agilent 1260 Infinity II system, equipped with UV detector, ELYSIA Raytest gamma radiation detector (Sockel 2 GABI Nova, Elysia S.A., Straubenhardt, Germany), thermostat for column and a manual injector was used to determine the radiochemical purity (RCP) of the radioactive solution. The chromatographic column used in this case was a column with a reverse-phase separation mechanism, Agilent Eclipse XDB-C18, 4.6 × 150 mm, with a particle size of 5 μm. The mobile phase was composed of water with TFA and acetonitrile. The analysis was performed in an isocratic gradient, with a flow rate of 3.5 mL/min.

Gamma-ray spectrometry method was used to determine the radioactive purity of the final solution using an HPGe detector from Baltic Scientific Instruments (Latvia). In order to determine the radioactive impurities and the characteristic ^64^Cu peaks (511 keV and 1345 keV), a sample of 2 μL of [^64^Cu]CuCl_2_ was evaluated 24 h from EOB, with a dead time of 0.7%.

The in vitro affinity of the radiolabelled compounds for target receptors on the surface of the cells was tested using the real-time receptor-ligand interaction monitoring system Ligand Tracer Yellow (LigandTracer Ridgeview Instruments, Uppsala, Sweden). The processed graphs were obtained using the dedicated TraceDrawer software, 1.6.1.

Three human tumours cell lines and a control normal cell line were tested: HT29 (colon adenocarcinoma, ATCC product, catalogue number HTB-38, from BIO ZYME S.R.L., Cluj-Napoca, Romania), HCT116 (colon carcinoma, ATCC product, catalogue number CCL-247, from BIO ZYME S.R.L., Cluj-Napoca, Romania), DU145 (prostate carcinoma, CLS product, catalogue number 300168, from CLS Cell Lines Service GmbH, Eppelheim, Germany), and BJ (normal human skin fibroblast, ATCC product, catalogue number CRL-2522, from BIO ZYME S.R.L., Cluj-Napoca, Romania) [23,24,25,26]. The cells were cultivated at 37 °C and 5% CO_2_ in a DMEM culture medium (Gibco, ThermoFisher Scientific, Waltham, MA, USA) supplemented with 10% FBS (Euroclone, Italy), and a stabilised Antibiotic Antimycotic Solution (100×) (Sigma, Saint Louis, MO, USA) containing 10,000 units penicillin, 10 mg streptomycin and 25μg Amphotericin B per mL. This culture medium will be further designated as a complete culture medium.

### 4.2. Target Preparation of Solid and Liquid Targets

#### 4.2.1. Preparation of Solid Target

A platinum well, which is part of the shuttle, was used for the electrodeposition of enriched ^64^Ni (99.53%). Platinum was chosen due to its chemical resistance to concentrated acids, allowing the use of high-concentrated HCl in the dissolution step of the irradiation target without affecting the support. The outer body is made of aluminium, chemically treated for anticorrosion, while the inner body is made of platinum. The platinum well (D16 × 12 mm) is pressurised into the aluminium body so that the shuttle assembly acts as a single component.

**Figure 5:**
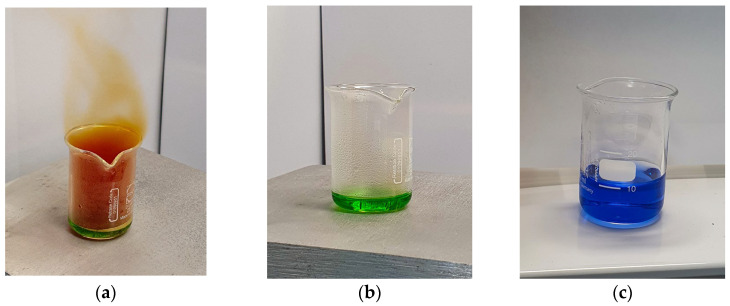
Preparation of ^64^Ni solution for electroplating: (**a**) nickel-64 dissolved in HNO_3_ (60%); (**b**) evaporation step (**c**) final solution, further used for electroplating.

The ^64^Ni solution for the two targets was obtained by dissolving 100 mg of enriched nickel-64 in concentrated HNO_3_ (60%), then evaporated to approximately 0.3 mL by heating. After evaporation, 2 mL of the NH_4_Cl–NH_4_OH buffer solution and water were added to a total volume of 10 mL. The pH of the final solution was adjusted to 9.30 ± 0.02 (at 25 °C) with NH_4_OH (Figure 5).

Half of the final solution was placed in the electrodeposition cell, part of the EDS module, where the electroplating was performed at a current of 37 ± 2 mA for a minimum of 15 to a maximum of 20 h to obtain a compact metallic target, without stalagmites or cracks. The electrodeposition process is completed when the blue solution becomes colourless.

#### 4.2.2. Preparation of Liquid Target Solution

The nickel-64 solution was prepared by dissolving 200 mg nickel-64 metal form (95% isotopic enrichment) in highly concentrated nitric acid (69%) at room temperature (RT). The resulting solution had to be evaporated and redissolved in 10 mM nitric acid multiple times to remove the excess of nitric acid from the original solution. The final irradiated solution (10 mM nitric acid) has a pH ranging from 1.2 to 1.7 in a total volume of 2.7 mL.

### 4.3. Cyclotrons and Irradiation

#### 4.3.1. Solid Target Irradiation

**Figure 6:**
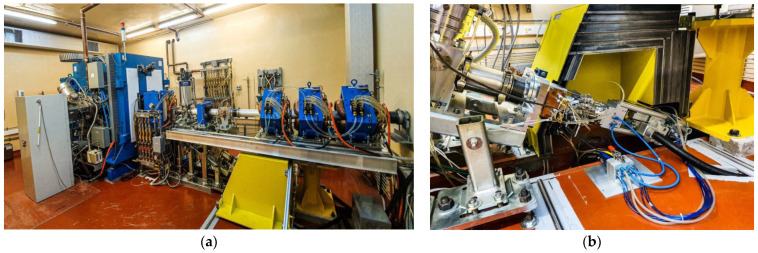
(**a**) TR-19 cyclotron and (**b**) the extension line and solid targets irradiation system.

The production of copper-64 was achieved by the irradiation of the resulted solid target on the TR-19 cyclotron (Advanced Cyclotron Systems Inc., Richmond, Canada), installed at the Radiopharmaceutical Research Centre, Horia Hulubei National Institute for Physics and Nuclear Engineering (IFIN-HH), Măgurele, Romania (Figure 6). TR-19 series cyclotrons are accelerators for negative hydrogen ions, with external ion sources. The energy range of the TR-19 proton beam, from 14 MeV to 19 MeV with a step of 100 keV, is ideal for most of the PET radioisotopes production. The energy of the extracted beam can be further degraded by using aluminium foils and Havar degradation windows, before reaching the target surface.

TR-19 can simultaneously extract two beams with intensities that vary independently, using two extraction ports that are diametrically opposed, to irradiate liquid, solid and gaseous targets, up to a total beam current of 300 μA. One of the ports can accommodate up to four reaction chambers, while the second extraction port is equipped with two external beam lines that can be switched remotely with a switching magnet [27].

The prepared target was sent to the solid target irradiation station installed on one of the extension lines of the TR-19 cyclotron and sloped down through the automated pneumatic transfer system (Figure 6b). Several simulations were performed in order to establish the optimal parameters of the irradiation. We used Geant4 for simulations of the nuclear reactions for ^64^Cu production, the code being implemented and validated, as described in our previous work [21]. Following the simulations, the irradiation parameters were set to an extracted energy of 14.2 ± 0.3 MeV, degraded to 11.81 ± 0.63 MeV on target by using a 320 μm thickness aluminium foil, with a beam current of 25 ± 0.5 μA and an irradiation beam time of 6 h.

#### 4.3.2. Liquid Target Irradiation

**Figure 7:**
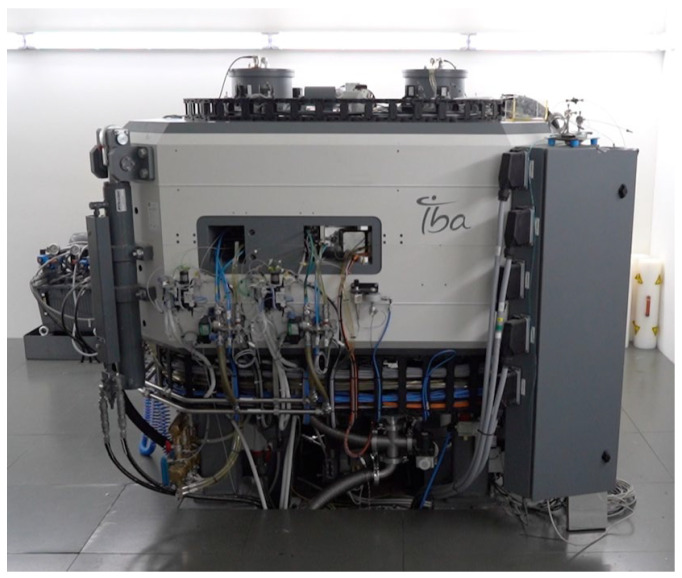
IBA Cyclone^®^ KIUBE.

The experiments were performed on the Cyclone^®^ KIUBE VE (IBA, Louvain-la-Neuve, Belgium) installed at the Institute for Nuclear Sciences Applied to Health (ICNAS), Coimbra, Portugal (Figure 7). This accelerator is a variable-energy cyclotron delivering protons with energy ranging from 13 to 18 MeV. With eight output ports, KIUBE is one of the most flexible systems, producing the widest range of radioisotopes used in PET imaging.

The liquid target system was loaded with 2.7 mL of the dissolved nickel-64 solution. The 18 MeV proton beam extracted was degraded down to 16.9 MeV on target by using a 75 μm thickness niobium window. The beam current was 50–55 μA and the irradiation time was adjusted according to the production needs.

### 4.4. Post-Irradiation Processing

#### 4.4.1. Solid Target Dissolution and Purification

**Figure 8:**
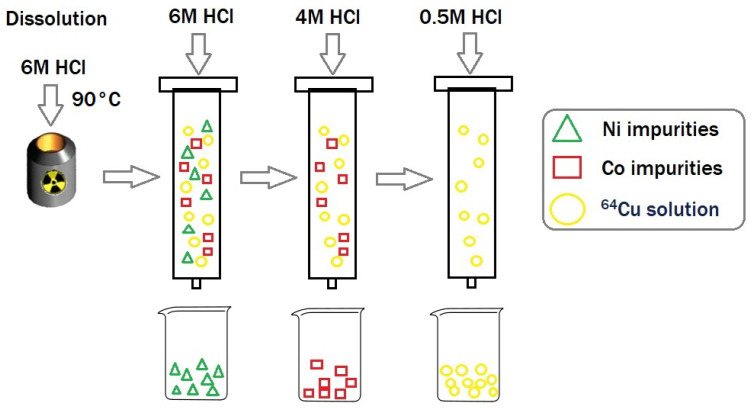
Dissolution and purification process after ST irradiation.

After irradiation, the shuttle was moved to the dissolution module using pneumatic transfer. The target was dissolved in the dissolution station of the EDS module using 2 × 1.5 mL of 6 M HCl while heating each portion for 15 min at 90 °C. The resulted crude solution was automatically transferred onto the separation column, installed in the purification module PRF. Each column was prepared approximately 1 h prior to use by loading 9 g of AG1-X8 resin. After dissolution, 30 mL of 6 M HCl were used to rinse without heating the dissolution cell and transfer tubes. These portions are also sent to the separation column to wash the nickel impurities. Next, 10 mL of 4 M HCl were used to wash Co impurities from the column. Finally, the copper-64 solution was eluted from the resin with 0.5 M HCl (Figure 8) in the form of [^64^Cu]CuCl_2_. The volume of the final solution can be selected depending on which parameter is of interest: higher amount of recovered activity (up to 19 mL) or higher specific activity (at least 3 mL), the latter variant also benefiting from a shorter processing time.

#### 4.4.2. Liquid Target Purification

After irradiation, the irradiated liquid target was automatically sent to the purification module (IBA Synthera^®^ Extension Module—Louvain-la-Neuve, Belgium), where the process lasted 30 min. The purification process is a two-step method using CU resin (1 mL) and SAX resin (2 mL), which were both preconditioned with water (10 mL)and 8 M HCl, respectively, before being used. The irradiated solution (V = 2.7 mL, pH ± 1.5) was loaded onto the CU resin (an oxime-based resin) from Triskem International (Bruz, France) after being diluted with water to a pH > 2.5. Next, the resin was washed with 1 mM HNO_3_ (10 mL) to ensure the complete removal of all cobalt and nickel ions. Finally, the column was eluted with 8 M HCl (2 mL). The solution eluted from CU resin was loaded onto a SAX resin (AG1X8—2 mL) to convert copper into a ready-to-use labelling solution.

### 4.5. Peptide Radiolabelling

The current study focused on the labelling of two neuropeptides used in molecular imaging applications and conjugated with the DOTA chelator: Neurotensin(8-13), DOTA-NT(8-13) and Neuromedin N, DOTA-NN. The binding of copper-64 to the peptide using a macrocyclic bifunctional chelator was performed at 95 °C, 20–30 min reaction time. For each 20 nmol of each peptide, 1–2 mL of [^64^Cu]CuCl_2_ solution (pH = 3.8–4.0, radioactive concentration 500–1500 MBq/mL) were added.

**Figure 9:**
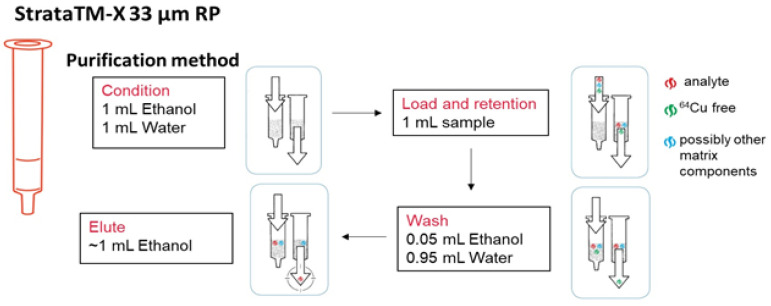
Radiolabelled peptide purification scheme.

After radiolabelling, the solution was purified using a Strata-X 33 µm RP cartridge, to separate the radiolabelled peptide from other impurities (Figure 9). The radiolabelled peptide was eluted with 1 mL of ethanol. The ethanol was further removed by evaporation at 80° C to near dryness. After evaporation, the radiolabelled peptide was recovered with 1 mL physiological solution, the pH was checked and adjusted, if needed, to 7.0–7.5.

### 4.6. In Vitro Uptake-Retention Assay

The method involves cell cultures (400,000 cells/sample) seeded in a marked defined area of a 91 mm Petri dish placed at a 30° angle, 24 h before analysis. During data acquisition, the Petri dish was placed in the device in the angled support and rotated periodically to detect the radioactivity in two situations: the cell with the medium and added radioisotope, and the medium with added radioisotope without cells. The calculated difference between the activities measured in these two situations allows for the evaluation of the quantity of radiolabelled compounds bonded to the cell receptors at regular intervals [28]. The acquired data are presented in a graph that indicates signal intensity (counts of radioactive decay per second) over time. The software takes into account the decay of the radioisotope during acquisition. Radiolabelled peptides affinity for cell receptors was analysed by adding a radioactive solution to the culture medium with an activity of 25 ± 5 MBq. The retention of the peptides in the cell lines was analysed by removing the medium, washing the cells with 1 mL of prewarmed complete culture medium, and adding 1 mL of fresh prewarmed complete culture medium.

## 5. Conclusions

Copper-64 can be produced in a high-quality [^64^Cu]CuCl_2_ solution using variable energy cyclotrons with a (p,n) reaction, and either solid or liquid nickel-64 targets. The radiochemical purity after post-irradiation processing reaches 99% (LT) and 99.99% (ST), respectively, while radionuclide purity is above 99.9%. The irradiation of a solid target yields to 117 MBq/μAh (17.6 ± 2.1 GBq/batch, decay corrected to EOB). The purification process is completed within 1 h using an automated processing module with a 90.90% activity yield. The resulted solution, with a high radioactive concentration (1340.4 ± 70.1 MBq/mL) was used for neuropeptide radiolabelling. The irradiation of a liquid target with 16.9 MeV protons yields to 12.4 MBq/μAh (3.7 ± 0.2 GBq/batch, decay corrected to EOB). Benefiting from a shorter purification process of only 40 min, the activity yield is similar at 90.87%, while the radioactive concentration of a radiolabelling solution is lower (492 MBq/mL). The [^64^Cu]CuCl_2_ solutions prepared by the two routes were successfully used for the radiolabelling of DOTA-NT(8-13) and DOTA-NN neuropeptides, resulting in high RCP (>99%), and high molar activity (27.2 and 26.4 GBq/μmol for LT route compared to 45 and 52 GBq/μmol for ST route, respectively). Their specificity for NTR is confirmed by the strong interaction between the [^64^Cu]Cu-DOTA-NT(8-13) and the colon cancerous cells lines HT29 and HCT116, as shown by the uptake-retention curves. Neurotensin (8-13) and Neuromedin N neuropeptides, will be further investigated as theranostic agents for cancers overexpressing neurotensin receptors.

## Figures and Tables

**Table 1:** Comparison of the outcomes obtained with different irradiation parameters on the solid target system for copper-64 production.

Parameters		Previous Settings [21]	New Settings
Proton energy (MeV) extracted/on target		14/11.6	14.2/11.8
Beam current (μA)		20	25
Beam time (hours)		4	6
Integrated beam current (μA·h)		80	150
Activity ^1^ (GBq)	Simulation	10.3 ± 0.5	21.6 ± 0.8
	Experiment	8.7 ± 0.7	17.6 ± 2.1

^1^ activity corrected to EOB.

**Table 2:** Summary of radiolabelling parameters for solid target vs. liquid target experiments.

	ST		LT	
Peptides	DOTA-NT(8-13)	DOTA-NN	DOTA-NT(8-13)	DOTA-NN
Quantity (nmol)	20		20	
Radiolabelling pH	3.9 ± 0.1		3.9 ± 0.1	
[^64^Cu]CuCl_2_ volume (mL)	1.00 ± 0.2		1.75 ± 0.25	
[^64^Cu]CuCl_2_ activity (MBq)	1340.4 ± 70.1		861.8 ± 17.3	
Temperature (°C)	95		95	
Reaction time (min)	25		25	
Radiolabelling yield (%)	67.04 ± 2.68	78.15 ± 3.12	63.24 ± 2.53	61.22 ± 2.45
Radiochemical purity (%)	99.99%	99.99%	99.08%	99.28%

## Data Availability

The original contributions presented in the study are included in the article, further inquiries can be directed to the corresponding authors.
